# Oral mucosa stem cells alleviates spinal cord injury-induced neurogenic bladder symptoms in rats

**DOI:** 10.1186/1423-0127-21-43

**Published:** 2014-05-13

**Authors:** Young-Sam Cho, Il-Gyu Ko, Sung-Eun Kim, Sung-Min Lee, Mal-Soon Shin, Chang-Ju Kim, Sang-Hoon Kim, Jun-Jang Jin, Khae-Hawn Kim

**Affiliations:** 1 Department of Urology, Kangbuk Samsung Hospital, Sungkyunkwan University School of Medicine, 29, Saemunan-ro, Jongno-gu, Seoul 110-746, Republic of Korea; 2 Department of Physiology, College of Medicine, Kyung Hee University, 26, Kyungheedae-ro, Dongdaemun-gu, Seoul 130-701 Republic of Korea; 3 Department of Physical Education, Graduate School of Education, Sangmyung University, 20 Hongjimun 2-gil, Jongno-gu, Seoul 110-743, Republic of Korea; 4 Department of Physical Activity Design, College of Science, Hanseo University, 46, Hanseo 1-ro, Haemi-myeon, Seosan 356-706, Republic of Korea; 5 Department of Urology, Gachon University Gil Medical Center, Gachon University School of Medicine, 21, Namdong-daero 774beon-gil, Namdong-gu, Incheon 405-760, Republic of Korea

**Keywords:** Spinal cord injury, Oral mucosa stem cells, Cystometry, Apoptosis, Nerve growth factor, c-Fos

## Abstract

**Background:**

Spinal cord injury (SCI) deteriorates various physical functions, in particular, bladder problems occur as a result of damage to the spinal cord. Stem cell therapy for SCI has been focused as the new strategy to treat the injuries and to restore the lost functions. The oral mucosa cells are considered as the stem cells-like progenitor cells. In the present study, we investigated the effects of oral mucosa stem cells on the SCI-induced neurogenic bladder in relation with apoptotic neuronal cell death and cell proliferation.

**Results:**

The contraction pressure and the contraction time in the urinary bladder were increased after induction of SCI, in contrast, transplantation of the oral mucosa stem cells decreased the contraction pressure and the contraction time in the SCI-induced rats. Induction of SCI initiated apoptosis in the spinal cord tissues, whereas treatment with the oral mucosa stem cells suppressed the SCI-induced apoptosis. Disrupted spinal cord by SCI was improved by transplantation of the oral mucosa stem cells, and new tissues were increased around the damaged tissues. In addition, transplantation of the oral mucosa stem cells suppressed SCI-induced neuronal activation in the voiding centers.

**Conclusions:**

Transplantation of oral mucosa stem cells ameliorates the SCI-induced neurogenic bladder symptoms by inhibiting apoptosis and by enhancing cell proliferation. As the results, SCI-induced neuronal activation in the neuronal voiding centers was suppressed, showing the normalization of voiding function.

## Background

Spinal cord injury (SCI) results in complete or incomplete loss of neuronal functions such as mobility and sensory functions [1]. SCI deteriorates various physical functions, in particular, bladder problems occur as a result of damage to the spinal cord. Following SCI, messages from the bladder and sphincter muscles dose not reach to the brain, which means that the affected person cannot feel when the bladder is full. This bladder dysfunction is termed as the “neurogenic bladder”. The signs of neurogenic bladder are urinary incontinence, inability to empty the bladder, urinary frequency, and urinary tract infections [2].

The ability of the lower urinary tract system to store and eliminate urine is controlled by a complex system of neural pathways. Bladder and external urethral sphincter are innervated from many areas of central nervous system, such as pontine micturition center (PMC), locus coeruleus, hypothalamus, preoptic area, and spinal cord [3]. The PMC plays an important role in the control of urinary bladder function, and PMC is known as the supraspinal switching center. It regulates the storage and elimination of urine [4]. The PMC is densely innervated by the medial preoptic nucleus (MPA). Two regions that maintain direct projections to the PMC are the periaqueductal gray matter (PAG) and the MPA of the hypothalamus [5]. The PAG-PMC projection is believed to take part in the micturition reflex. Neurons in the PAG are known to regulate the micturition reflex, and lesions in the PAG cause severe urinary dysfunction [6].

The transcription factor c-Fos is encoded by the immediate early gene c-Fos, and c-Fos expression has been used as a marker of neuronal activity [7,8]. Stimulation of the urinary bladder increased the number of c-Fos-immunoreactive neurons in the PAG, PMC, and spinal cord [4,9]. After SCI, the neuronal activity in the neuronal voiding tracts was increased [10,11]. Another important parameter representing neuronal activation in the voiding centers is nerve growth factor (NGF) [12]. NGF is produced by urothelium and smooth muscle cells [13]. NGF is implicated in the pathogenesis of urinary bladder overactivity at the spinal level, and NGF modulates the neuronal function *via* the micturition reflex pathway [14].

Treatments of neurogenic bladder caused by SCI include physical-psychological method, electrical-stimulatory method, chemotherapy, and surgery [2,15]. However, these methods have some side effects and sometimes resulted in incomplete recovery. Moreover, there is no gold standard in the treatment of patients with neurogenic bladder symptoms without the treatment of SCI. Stem cell transplantation is one of the most promising fields for spinal cord regeneration, because stem cells can achieve regeneration of the injured spinal cord by replacing the damaged neuronal tissues [16,17]. In particular, oral mucosa stem cells can be extracted in a simple and reliable manner. Oral mucosa stem cells can trans-differentiate into functional neural cells, and these cells have low immunogenicity [18,19].

The possibility that oral mucosa stem cells can be used for the central nervous repair has been raised, however the efficacy of oral mucosa stem cells on the recovery of neurogenic bladder following SCI is not clearly documented. In the present study, we investigated the effects of oral mucosa stem cells on the SCI-induced neurogenic bladder in relation with apoptotic neuronal cell death and cell proliferation. In this study, cystometry, hematoxylin and eosin (H & E) staining, terminal deoxynucleotidyl transferase-mediated dUTP nick end labeling (TUNEL) staining were conducted. Immunofluorescence for smooth muscle actin-α (SMA-α) and Ki67 were performed. Neuronal activation was assessed by immunohistochemistry for c-Fos and NGF in the neuronal voiding centers (MPA, PAG, and PMC spinal cord L4-L5).

## Methods

### Experimental animals and treatment

Adult male Sprague-Dawley rats, weighing 260 ± 10 g (13 weeks), were used in this experiment. The experimental procedures were performed in accordance with the animal care guidelines of the National Institutes of Health (NIH) and the Korean Academy of Medical Sciences. The rats were housed under controlled temperature (23 ± 2°C) and lighting (08:00 to 20:00 h) conditions with food and water available *ad libitum*. The rats were randomly divided into three groups (n = 10 in each group), as follows: the sham-operation group, the SCI-induced group, and the SCI-induced and oral mucosa stem cell transplantation group.

### Primary culture of oral mucosa cells

Specimens were obtained from the oral mucosa membrane of the rats (weight: 260 ± 10 g; age: 13 weeks) within 1 hour after surgical resection. The isolated tissues were cut into 1–2 mm three pieces, and washed with Ca_2_1-Mg_2_1-free Dulbecco’s PBS (DPBS), enzymatically digested for 1 hour at 37°C with 3 mg/ml of collagenase type I. The samples were filtered using 40 μm cell strainers and centrifuged at 1,300 rpm for 3 min; the pellets were collected as cells. The cells were maintained in low-glucose DMEM supplemented with 10% heat-inactivated FBS, 1% penicillin/streptomycin, and 1% gentamycin. The cells of the same group were pooled together for all experiments.

### Induction of spinal cord injury and oral mucosa stem cell transplantation

For the induction of SCI and the transplantation of oral mucosa stem cells, the rats were anesthetized by inhalation of isoflurane (2% isoflurane in 30% O_2_ and 70% N_2_, JW Pharmaceutical Corporation, Kyung-Gi, Korea) during surgery. The skin in the T10-T12 areas was incised through the 2.5 cm median incision, and the thoracic vertebral column and the spinous process in T11 were exposed by dissection. After dissection, the spinous process in T11 was approached by using a drill up to the vertebral arch area, and 2 mm deep hole was made from the surface of the vertebral arch by using a surgical drill (diameter: 1 mm), and the damage to the spinal cord was produced by using a 22 gauge needle. Then, 100 μl of oral mucosa stem cells was infused over the course of 1 min by using a 22-gauge insert vein (IV) catheter (ETFE0120, Sewon Med Co. Ltd, Seoul, Korea). The IV catheter remained in place for an additional 3 min after the infusion and was subsequently withdrawn; the hole was then sealed. The skin was closed layer by layer. The body temperature was maintained at 36 ± 0.5°C during the surgery using a homeothermic blanket control unit (Harvard Apparatus, Massachusetts, MA) that enveloped the body and the head. After recovery, the animals were monitored for an additional 2 hours to prevent hypothermia.

### Cystometry

Bladder function was evaluated by cystometry on 21 days after induction of SCI, as the previously described method [8,20]. The rats were anesthetized with Zoletil 50® (10 mg/kg, i.p.; Vibac Laboratories, Carros, France). A sterile polyethylene catheter (PE50) was inserted into the urethra through the bladder dome. The catheter was connected to a pressure transducer (Harvard Apparatus, Holliston, MA) and a syringe pump (Harvard Apparatus) *via* a three-way stopcock to record intravesical pressure and to infuse saline into the bladder. After the bladder was emptied, cystometry was performed with an infusion of 0.5 ml saline. The contraction pressure and the contraction time in the bladder were monitored using LabScribe (iWork System Inc., Dover, NH).

### Tissue preparation

The rats were sacrificed immediately after determining the contraction pressure and the contraction time. The animals were anesthetized using Zoletil 50® (10 mg/kg, i.p.; Vibac Laboratories), transcardially perfused with 50 mM phosphate-buffered saline (PBS), and fixed with a freshly prepared solution consisting of 4% paraformaldehyde in a 100 mM phosphate buffer (PB, pH 7.4). The brains and spinal cords were dissected and postfixed in the same fixative overnight, and then transferred into a 30% sucrose solution for cryoprotection. In the brains, the 40 μm thick coronal sections and the 20 μm thick transverse section in the spinal cord were made using a freezing microtome (Leica, Nussloch, Germany). Ten slice sections, on average, from each region were collected from each rat. For the recovery of SCI, the spinal cord was selected from the region spanning from T10 to T12. Furthermore, the PMC was selected from the region spanning from Bregma −9.68 to −9.80 mm; the ventrolateral PAG (vlPAG) was selected from the region spanning from Bregma −7.64 to −8.00 mm; the MPA was selected from the region spanning from Bregma −0.26 to 0.80 mm; and the spinal cord was selected from the L4-L5 regions.

### Hematoxylin and eosin staining

To detect histological changes in the spinal cord tissues, H & E staining was performed. The slides were dipped into Mayer’s hematoxylin for 30 sec, rinsed with tap water until they were clear, dipped in eosin for 30 sec, and again rinsed with water. The slides were air-dried at room temperature and then, dipped twice in 95% ethanol, twice in 100% ethanol, twice in a solution of 50% ethanol and 50% xylene, and twice in 100% xylene. The coverslips were finally mounted using Permount® (Fisher Scientific, New Jersey, NJ).

### TUNEL assay

To visualize the DNA fragmentation, a marker of apoptotic cell death, TUNEL staining was performed, as the previously described method [21] using an *in situ* cell death detection kit® (Roche, Mannheim, Germany). The sections were post-fixed in ethanol-acetic acid (2:1) and rinsed. Then, the sections were incubated with proteinase K (100 μg/ml), rinsed, incubated in 3% H_2_O_2_, permeabilized with 0.5% Triton X-100, rinsed again, and incubated in the TUNEL reaction mixture. The sections were rinsed and visualized using Converter-POD with 0.03% 3,3′-diaminobenzidine (DAB). Mayer’s hematoxylin (DAKO, Glostrup, Denmark) was used for counter-staining, and the sections were finally mounted onto gelatin-coated slides. The slides were air-dried overnight at room temperature, and the coverslips were mounted using Permount® (Fisher Scientific).

### Immunofluorescence assay

Immunofluorescence assay was conducted for the detection of SMA-α and Ki67, as the previously described method [22]. For dual fluorescence labeling, the fixed tissues were incubated at 36°C for 2 hours with the following antibodies: mouse monoclonal anti-SMA-α (1:100, M0851, Dako, Seoul, Korea) and rabbit polyclonal to active anti-Ki67 (1:100, H-300, Santa Cruz Biotechnology, Santa Cruz, CA). After washing with PBS, both cell types were incubated at room temperature for 1 hour with Alexa Fluor 488 goat anti-mouse IgG and Texas Red goat anti-rabbit IgG antibodies (1:1000, A10677, T2767, Invitrogen, Eugene, OR). PBS contained 1% normal horse serum and Triton X-100 for double localization. After three further washes with PBS, the slides were cover-slipped with a Vectashield medium (H-1200, Vector Laboratories, Burlingame, CA).

### Immunohistochemistry for c-Fos and NGF

c-Fos and NGF expressions were determined by immunohistochemistry, as the previously described method [8]. Free-floating tissue sections were incubated overnight with rabbit anti-c-Fos and mouse anti-NGF antibodies (Santa Cruz Biotechnology) at a dilution of 1:1000, and the sections were then incubated for 1 hour with biotinylated anti-rabbit and anti-mouse secondary antibodies (Vector Laboratories). The sections were subsequently incubated with an avidin-biotin-peroxidase complex (Vector Laboratories) for 1 hour at room temperature. Immunoreactivity was visualized by incubating the sections in a solution consisting of 0.05% DAB and 0.01% H_2_O_2_ in a 50-mM Tris buffer (pH 7.6) for approximately 3 min. The sections were then washed three times with PBS and mounted onto gelatin-coated slides. The slides were air-dried overnight at room temperature, and the coverslips were mounted by using Permount® (Fisher Scientific).

### Data analysis

Images of SMA-α and Ki67 in the spinal cords were collected using a Zeiss LSM 700 confocal microscope (Carl Zeiss MicroImaging Inc., Jena, Germany). A confocal fluorescence image stimulated by 488-nm and 568-nm lasers was filtered by using a green emission filter and a red/blue dual-emission filter, respectively. The numbers of TUNEL-positive cells in the spinal cord and the numbers of c-Fos and NGF-positive cells in the neuronal voiding centers (MPA, vlPAG, PMC, and spinal cord L4-L5) were counted hemilaterally through a light microscope (Olympus, Tokyo, Japan). The area of the spinal cord and neuronal voiding centers from each slice was measured using an Image-Pro® Plus computer-assisted image analysis system (Media Cybernetics Inc., Silver Spring, MD) attached to a light microscope (Olympus, Tokyo, Japan).

Statistical analysis was performed by using one-way analysis of variance (ANOVA) followed by Duncan’s post-hoc test, and the results were expressed as the mean ± standard error of the mean (SEM). The significance was set as *P* < 0.05.

## Results

### Effects of oral mucosa stem cells on the contraction pressure and the contraction time in the urinary urinary bladder

**Figure 1:**
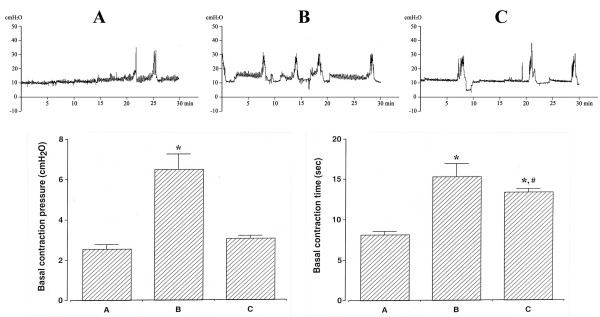
**Effects of transplantation of oral mucosa stem cells on contraction pressure and time in cystometry.** Upper: Graphs of cystometry in each group. Lower: Analysis of contraction pressure (left) and contraction time (right). **(A)** Sham-operation group, **(B)** SCI-induced group, and **(C)** SCI-induced and oral mucosa stem cell transplantation group. * represents *P* < 0.05 as compared to the sham-operation group. # represents *P* < 0.05 as compared to the SCI-induced group.

The contraction pressure was 2.55 ± 0.22 cmH_2_O in the sham-operation group, 6.49 ± 0.77 cmH_2_O in the SCI-induced group, and 3.05 ± 0.14 cmH_2_O in the SCI-induced and oral mucosa stem cell transplantation group. The contraction time was 8.09 ± 0.43 sec in the sham-operation group, 15.29 ± 1.65 sec in the SCI-induced group, and 13.36 ± 0.40 sec in the SCI-induced and oral mucosa stem cell transplantation group (Figure 1).

These results showed that the contraction pressure and the contraction time in the urinary bladder were increased by induction of SCI (*P* < 0.05), whereas transplantation of oral mucosa stem cells into the insult area alleviated the SCI-induced contraction pressure and contraction time (*P* < 0.05).

### Effect of oral mucosa stem cells on the histological alterations in the spinal cord tissues

**Figure 2:**
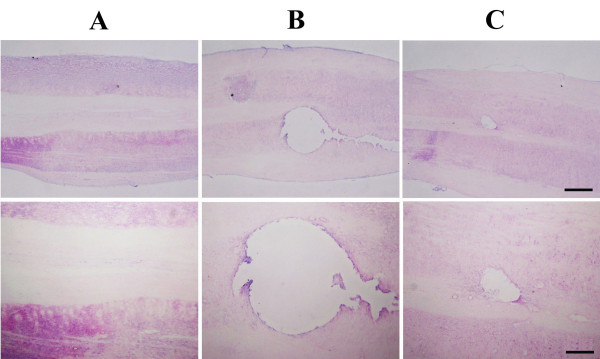
**Effects of oral mucosa stem cell transplantation on histological alterations in the spinal cord tissues.** The scale bar represents 40 μm in upper panels and 150 μm in lower panels. **(A)** Sham-operation group, **(B)** SCI-induced group, and **(C)** SCI-induced and oral mucosa stem cell transplantation group.

The normal spinal cord was observed in the sham-operation group. In the SCI group, H & E staining showed the completely disrupted lesion in the dorsal area. However, transplantation of oral mucosa stem cells decreased the SCI-induced disrupted lesion, and new tissues were increased around the damaged tissues (Figure 2).

### Effect of oral mucosa stem cells on the number of TUNEL-positive cells in the spinal cord tissues

**Figure 3:**
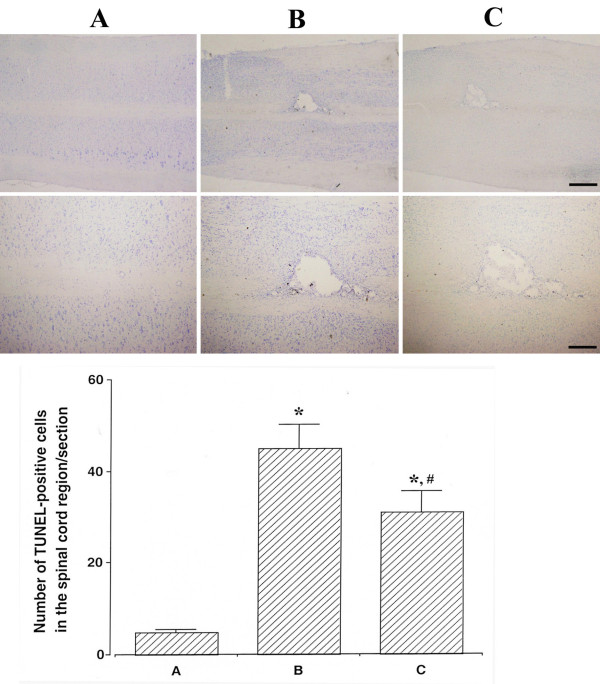
**Effects of transplantation of oral mucosa stem cells on DNA fragmentation in the spinal cord tissues.** Upper: Photomicrographs of terminal deoxynucleotidyl transferase-mediated dUTP nick end labeling (TUNEL)-positive cells in the spinal cord tissues. The scale bar represents 40 μm in upper panels and 150 μm in lower panels. Lower: Number of TUNEL-positive cells in each group. **(A)** Sham-operation group, **(B)** SCI-induced group, and **(C)** SCI-induced and oral mucosa stem cell transplantation group. * represents *P* < 0.05 as compared to the sham-operation group. # represents *P* < 0.05 as compared to the SCI-induced group.

The number of TUNEL-positive cells was 4.84 ± 0.62/section in the sham-operation group, 44.92 ± 5.21/section in the SCI-induced group, and 30.92 ± 4.60/section in the SCI-induced and oral mucosa stem cell transplantation group (Figure 3).

These results showed that apoptosis was increased by induction of SCI (*P* < 0.05), whereas transplantation of the oral mucosa stem cells suppressed the SCI-induced apoptosis (*P* < 0.05).

### Effect of oral mucosa stem cells on the SMA-α and Ki67 expressions in the spinal cord tissues

**Figure 4:**
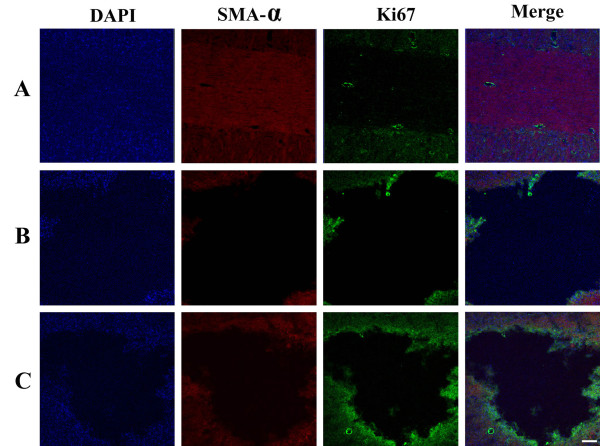
**Effects of transplantation of oral mucosa stem cells on smooth muscle actin-α (SMA-α) and Ki67 expressions in the spinal cord tissues. (A)** Sham-operation group, **(B)** SCI-induced group, and **(C)** SCI-induced and oral mucosa stem cell transplantation group. The scale bar represents 150 μm.

The photomicrographs showed that transplantation of oral mucosa stem cells increased the SMA-α and Ki67 expressions as compared to those in the SCI-induced group. Moreover, the SCI-induced lesion area was decreased by transplantation of oral mucosa stem cells, and new tissues were increased around the damaged tissues (Figure 4).

### Effect of oral mucosa stem cells on the number of c-Fos-positive cells in the neuronal voiding centers

**Figure 5:**
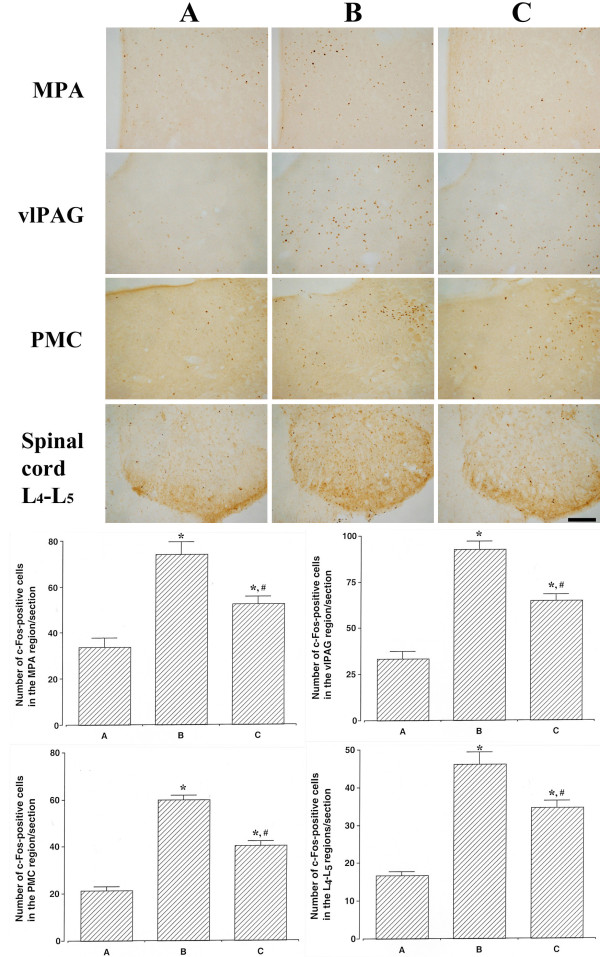
**Effects of transplantation of oral mucosa stem cells on c-Fos expressions in the neuronal voiding centers.** Upper: Photomicrographs of c-Fos-positive cells in the neuronal voiding centers. The scale bar represents 200 μm. MPA: pontine micturition center, vlPAG: ventrolateral periaqueductal gray matter, PMC: pontine micturition center, L4-L5: Lumbar 4-Lumbar 5. Lower: Number of c-Fos-positive cells in each group. **(A)** Sham-operation group, **(B)** SCI-induced group, and **(C)** SCI-induced and oral mucosa stem cell transplantation group. * represents *P* < 0.05 as compared to the sham-operation group. # represents *P* < 0.05 as compared to the SCI-induced group.

The number of c-Fos-positive cells in the MPA region was 33.69 ± 4.15/section in the sham-operation group, 74.07 ± 5.43/section in the SCI-induced group, and 52.46 ± 3.16/section in the SCI-induced and oral mucosa stem cell transplantation group. The number of c-Fos-positive cells in the vlPAG region was 33.38 ± 2.37/section in the sham-operation group, 92.61 ± 4.10/section in the SCI-induced group, and 64.84 ± 4.30/section in the SCI-induced and oral mucosa stem cell transplantation group. The number of c-Fos-positive cells in the PMC region was 21.46 ± 1.45/section in the sham-operation group, 60.00 ± 1.87/section in the SCI-induced group, and 40.38 ± 1.97/section in the SCI-induced and oral mucosa stem cell transplantation group. The number of c-Fos-positive cells in the spinal cord L4-L5 regions was 16.69 ± 0.97/section in the sham-operation group, 46.15 ± 3.21/section in the SCI-induced group, and 34.69 ± 1.73/section in the SCI-induced and oral mucosa stem cell transplantation group (Figure 5).

These results showed that the c-Fos expression in the neuronal voiding centers was increased by induction of SCI (*P* < 0.05). However, transplantation of oral mucosa stem cells decreased the SCI-induced c-Fos expression in the neuronal voiding centers (*P* < 0.05).

### Effect of oral mucosa stem cells on the number of NGF-positive cells in the neuronal voiding centers

**Figure 6:**
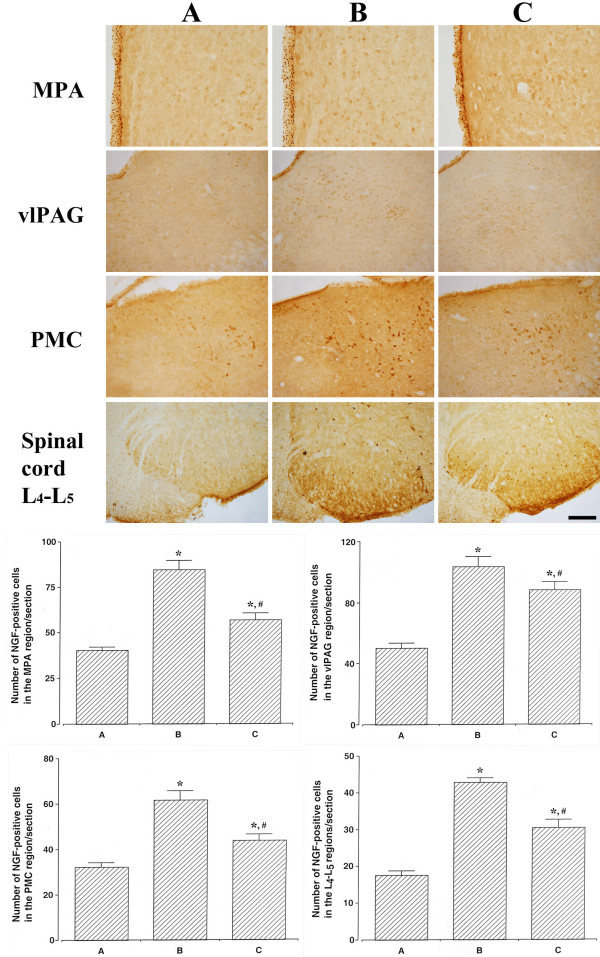
**Effects of transplantation of oral mucosa stem cells on nerve growth factor (NGF) expressions in the neuronal voiding centers.** Upper: Photomicrographs of NGF-positive cells in the neuronal voiding centers. The scale bar represents 200 μm. MPA: pontine micturition center, vlPAG: ventrolateral periaqueductal gray matter, PMC: pontine micturition center, L4-L5: Lumbar 4-Lumbar 5. Lower: Number of NGF-positive cells in each group. **(A)** Sham-operation group, **(B)** SCI-induced group, and **(C)** SCI-induced and oral mucosa stem cell transplantation group. * represents *P* < 0.05 as compared to the sham-operation group. # represents *P* < 0.05 as compared to the SCI-induced group.

The number of NGF-positive cells in the MPA region was 40.61 ± 1.65/section in the sham-operation group, 84.53 ± 4.97/section in the SCI-induced group, and 56.76 ± 3.77/section in the SCI-induced and oral mucosa stem cell transplantation group. The number of NGF-positive cells in the vlPAG region was 50.15 ± 3.05/section in the sham-operation group, 103.38 ± 6.49/section in the SCI-induced group, and 88.15 ± 5.40/section in the SCI-induced and oral mucosa stem cell transplantation group. The number of NGF-positive cells in the PMC region was 32.15 ± 2.06/section in the sham-operation group, 61.84 ± 3.88/section in the SCI-induced group, and 43.69 ± 2.88/section in the SCI-induced and oral mucosa stem cell transplantation group. The number of NGF-positive cells in the spinal cord L4-L5 regions was 17.53 ± 1.17/section in the sham-operation group, 42.76 ± 1.23/section in the SCI-induced group, and 30.46 ± 2.09/section in the SCI-induced and oral mucosa stem cell transplantation group (Figure 6).

These results showed that the NGF expression in the neuronal voiding centers was increased by induction of SCI (*P* < 0.05). However, transplantation of oral mucosa stem cells decreased the SCI-induced NGF expression in the neuronal voiding centers (*P* < 0.05).

## Discussion

Among many experimental SCI models, laminectomy and spinal cord contusion model is the oldest and the most widely used SCI animal model [23,24]. This model elicits sensory dysfunctions, including neuropathic pain, tactile allodynia, and thermal hyperalgesia similar to that of the SCI patients [23]. However, this model shows low survival rate due to the various side effects of surgery and has difficulty in the direct drug administration. In the present study, we established the needle-using injury model following many pilot studies. The advantage of this model is high survival rate, because of the rapid operation and minimum injury site. By this method, 100% survival rate was achieved and direct administration of the stem cells was possible without reflux in this study.

Following SCI, apoptosis plays an important role in the progression of the secondary sequelae [25]. The important biochemical feature of apoptosis is internucleosomal DNA fragmentation, and TUNEL assay detects the DNA fragmentation [26]. In the previous studies, DNA fragmentation was increased for hours or days following SCI [25,27]. In the present study, the number of TUNEL-positive cells was increased after SCI induction. In contrast, transplantation of the oral mucosa stem cells decreased the number of TUNEL-positive cells in the SCI-induced rats. The present results indicated that induction of SCI initiated apoptosis in the spinal cord tissues, whereas treatment with oral mucosa stem cells suppressed the SCI-induced apoptosis.

The expression of the Ki67 is strictly associated with new cell proliferation [22]. In oral epithelial tissues, increment of Ki67 expression represents enhancement of proliferating cells, which is relevant to the facilitation of tissue repair [28]. In the present study, the expression of Ki67 in the spinal cord was decreased after induction of SCI. Transplantation of oral mucosa stem cells increased Ki67 expression in the SCI rats. The present results indicated that effective proliferation of oral mucosa cells in the damaged spinal cord was achieved by transplantation of the oral mucosa stem cells.

Normal detrusor muscle allows bladder filling during the storage phase, with little or no change in the bladder pressure [29]. Sphincter remains closed during the increase in the intra-abdominal pressure, and involuntary bladder contraction does not appear [30]. In contrast, neurogenic bladder leads to involuntary detrusor muscle contraction and causes increased bladder pressure and prolonged contraction time [31]. The present study showed that the contraction pressure and the contraction time were increased after induction of SCI, indicating that SCI resulted in a neurogenic bladder. Transplantation of the oral mucosa stem cells decreased the contraction pressure and the contraction time in the SCI-induced rats. The present results indicated that neurogenic bladder symptoms were alleviated by transplantation of the oral mucosa stem cells.

Electrical or chemical stimulation on the lower urinary tract changed neuronal activity in the micturition centers, such as the PMC, PAG, MPA, and spinal cord [12,32]. In particular, injuries to the spinal cord overexpress the early genes in the bladder and PMC, because of a compensatory response with the disconnection of voiding tracts [33]. The previous studies reported that spinal cord damage caused overactive bladder symptoms and also increased c-Fos expression in the voiding centers [10,11]. Enhanced c-Fos expression in the voiding centers represented neuronal activation by overactive bladder [8,20]. The overexpression of NGF in the bladder and urethra is associated with the modulation disability of micturition in the neurogenic bladder symptoms caused by SCI [34,35]. Likewise, NGF expression in the voiding centers was also increased by stress urinary incontinence and overactive bladder [12,20]. In the present study, c-Fos and NGF expressions in the neuronal voiding centers were increased after SCI, indicating that the induction of SCI activated neurons in the voiding centers. It can be inferred that dysfunction of the nerve connection caused by SCI might strongly stimulate the micturition-related neuronal voiding centers in the brain. Expressions of c-Fos and NGF in the neuronal voiding centers were suppressed by transplantation of the oral mucosa stem cells. The present results suggested that transplantation of the oral mucosa stem cells suppressed SCI-induced neuronal activation in the voiding centers.

## Conclusions

This study showed that transplantation of oral mucosa stem cells ameliorated the SCI-induced neurogenic bladder symptoms by inhibiting apoptosis and by enhancing cell proliferation of the oral mucosa stem cells. As the results, SCI-induced neuronal activation in the neuronal voiding centers was suppressed, showing the normalization of voiding function. Our study revealed that oral mucosa stem cells showed effectiveness on the recovery of neurogenic bladder induced by SCI.

## Abbreviations

DAB: 3,3′-diaminobenzidine; H & E: Hematoxylin and eosin; MPA: Preoptic nucleus; NGF: Nerve growth factor; PAG: Periaqueductal gray matter; PBS: Phosphate-buffered saline; SCI: Spinal cord injury; SMA-α: Smooth muscle actin-α; TUNEL: Terminal deoxynucleotidyl transferase-mediated dUTP nick end labeling; vlPAG: ventrolateral periaqueductal gray matter.

## Competing interests

The authors declare that they have no competing interests.

## Authors’ contributions

K-HK conceived and designed this study; Y-SC drafted the manuscript and acquired data; C-JK supervised this study and provided the critical revision of the manuscript for important intellectual content; Il-GK, S-EK, and S-ML analyzed and interpreted data; M-SS, S-HK, and J-JJ provided the administrative, technical, or material support. All authors read and approved the final manuscript.
